# Antifungal and anti-inflammatory potential of the endangered aromatic plant *Thymus albicans*

**DOI:** 10.1038/s41598-020-75244-w

**Published:** 2020-11-02

**Authors:** Mariana Roxo, Mónica Zuzarte, Maria José Gonçalves, Jorge M. Alves-Silva, Carlos Cavaleiro, Maria Teresa Cruz, Lígia Salgueiro

**Affiliations:** 1 Institute of Pharmacy and Molecular Biotechnology (IPMB), Heidelberg University, Heidelberg, Germany; 2 Faculty of Medicine, Coimbra Institute for Clinical and Biomedical Research (iCBR), University of Coimbra, Coimbra, Portugal; 3 Centre for Innovative Biomedicine and Biotechnology (CIBB), University of Coimbra, Coimbra, Portugal; 4 Clinical Academic Centre of Coimbra (CACC), Coimbra, Portugal; 5 Faculty of Pharmacy of the University of Coimbra, University of Coimbra, Coimbra, Portugal; 6 Chemical Process Engineering and Forest Products Research Centre (CIEPQPF), Department of Chemical Engineering, Faculty of Sciences and Technology, University of Coimbra, Coimbra, Portugal; 7 Center for Neuroscience and Cell Biology (CNC), Coimbra, Portugal

**Keywords:** Drug discovery, Plant sciences

## Abstract

*Thymus albicans* is an endemic species of the Iberian Peninsula with a vulnerable conservation status. In an attempt to contribute to the valorization of this species, the present study brings new insights on the antifungal and anti-inflammatory mechanism of action of *T. albicans* essential oil. The antifungal activity of the oil and its major compounds was assessed for the first time against standard and clinically isolated strains of yeasts and filamentous fungi. The effect on the two major virulence factors of *Candida albicans* (germ tube formation and biofilm disruption) was considered in more detail. At 0.08 μL/mL, the oil inhibited *C. albicans* germ tube formation by more than 40% and decreased biofilm biomass at MIC values, thus pointing out its antivirulent potential. The anti-inflammatory activity of the essential oil was investigated on LPS-stimulated mouse macrophages (RAW 264.7) by evaluating the levels of several pro-inflammatory mediators, namely nitric oxide (NO), inducible nitric oxide synthase (iNOS) and cyclooxygenase-2 (COX-2). *T. albicans* oil reduced the production of nitrites, a NO derived sub-product, at non-cytotoxic concentrations of 0.32 and 0.64 μL/mL, by 27 and 41%, respectively. In addition, the iNOS protein levels of essential oil pre-treated cells were reduced by 14%. Overall, the high essential oil yield of *T. albicans* as well as its bioactive effects at concentrations without cytotoxicity, encourage further studies on the potential pharmacological applications of this species. Furthermore, these results raise awareness for the need to preserve endangered species that may hold relevant medicinal value.

## Introduction

Thyme species are among the most important medicinal and aromatic crops in non-tropical environments^1^. The medicinal and non-medicinal uses of these plants are mainly related to their volatile extracts. Several *Thymus* spp. extracts are widely used for the treatment of gastrointestinal, respiratory and skin disorders, and their use is regulated by some entities and agencies, such as the European Medicines Agency^2^, European Scientific Cooperative on Phytotherapy^3^, German Commission E and World Health Organization^4,5^.

Food and flavour industries take advantage of the aromatic and antiseptic properties of thyme essential oils applying them in the preservation of food products and in the production of cosmetics and toiletries^6^. Moreover, due to its aromatic characteristics, thyme is a daily used culinary herb all over the world.

*Thymus albicans* Hoffm. and Link, popularly known in Portugal as tomilho-alvadio [syn. *Origanum albicans* (Hoffmans. and Link) Kuntze; *Thymus mastichina* var. *micranthus* Boiss.; *Thymus tomentosus* var. *virescens* Coss.; *Thymus virescens* (Coss.) Pau]^7^ is an endemic species to the coastal area of the south-west Iberian Peninsula currently listed as vulnerable (meaning at high risk of extinction) by the IUCN Red List of Threatened Species^8^. It can only be found in the region of Algarve in Portugal, and in the provinces of Sevilla and Cádiz in Spain, being presently distributed in disperse and severely fragmented populations with a decreasing trend^9^. The main threats are related to the pressure exerted by mass tourism, agriculture and aquaculture practices.

*Thymus albicans* belongs to Sect. Mastichina, together with *Thymus mastichina* (L.) L*.* subsp. *mastichina* and *Thymus mastichina* subsp. *donyanae* R. Morales ^10^. The aerial flowering parts of the plant are traditionally used for the same purposes of *T. mastichina* subsp. *mastichina*, mainly for the treatment of oral and pharyngeal inflammation and dermatitis^11^. Additionally, there is ethnopharmacological evidence of its use as a stomach tonic in Spain^12^.

*Thymus mastichina* is one of the most industrially relevant thyme species, currently employed in the production of cleaning products, perfumes, fragrances, soaps and other cosmetics^13,14^. It is widely cultivated in Portugal and Spain, and has an ISO standard (4728:2003) specifying the quality parameters of the essential oil for industrial and commercial uses^15^. Several pharmacological activities have been reported for *T. mastichina* essential oil, including antimicrobial activity^16–19^, anti-inflammatory potential^20,21^, antioxidant activity^22–24^ and cytotoxicity against cancer cell lines^25–27^. Due to the similarities between the chemical profile of *T. mastichina* and *T. albicans* essential oils, it seems reasonable to assume that both species might possess similar bioactive properties. This assumption is in part corroborated by previous studies showing the antibacterial activity of *T. albicans* essential oil against Gram-positive bacteria^28^, its antioxidant activity^29,30^, its capacity to scavenge nitric oxide and to inhibit the activity of lipoxygenase in vitro, both commonly used as markers of anti-inflammatory potential^21^. As far as we know, those are the only studies on the bioactive potential of *T. albicans* essential oil. More studies are needed to further confirm the reported effects and to explore new bioactivities.

In this regard, the present study aimed to investigate the chemical profile, and the antifungal and anti-inflammatory activities of *Thymus albicans* essential oil from Portugal and its major compounds, concomitantly highlighting the mechanisms of action underlying these effects. These bioactivities were selected taking into account their current relevance in the clinic. In fact, invasive fungal infections are an increasingly important global health burden accounting for 1.66 million deaths every year^31^. While these mainly affect immunocompromised individuals, superficial fungal infections are reported to affect between 20 and 25% of the world’s population, including immunocompetent individuals^32^. Treatment failure and high relapse rates are mainly correlated with the extensive use of a limited number of non-selective antifungal drugs (e.g. fluconazole, amphotericin B and terbinafine) which leads to off-target toxicity and to the emergence of resistance among the main etiologic agents^33^. Chronic inflammation is another factor that contributes to the successful colonization of the host tissues by pathogenic fungi and may impede disease eradication^34^. Taken together, these facts justify the urgency for therapeutic alternatives, preferentially combining selective antifungal and anti-inflammatory activities.

## Results and discussion

### Essential oil composition

Prior to this study, several essential oils from *T. albicans* plants collected in different regions of Algarve (Portugal) were chemically characterized, revealing chemical variability regarding the four major compounds 1,8-cineole (29–43%), linalool (5–25%), α-terpineol (4.5–9.8%) and borneol (2.2–8.0%). The most predominant chemical profile was characterized by high contents of 1,8-cineole and linalool. Therefore, for the present study we selected the sample that better represented the chemical profile of the essential oils from Portuguese *T. albicans*. This sample was obtained from populations with the same chemical profile growing in Foz de Almargem coastal lagoon in Algarve. The chemical characterization of the obtained essential oil is shown in Table 1.

**Table 1:** Composition of the essential oil of *Thymus albicans.*

RI^a^	RI^p^	Compounds^a^	%
922	1030	α-Thujene	0.2
930	1030	α-Pinene	1.7
943	1073	Camphene	2.3
964	1128	Sabinene	1.1
970	1118	β-Pinene	1.9
980	1161	Myrcene	0.7
1006	1185	α-Terpinene	0.4
1013	1272	*p*-Cymene	0.1
1020	1206	Limonene	0.6
1020	1212	1,8-Cineole	40.5
1035	1250	*trans*-β-Ocimene	0.2
1047	1250	*γ-*Terpinene	0.3
1050	1459	*trans*-Sabinene hydrate	0.1
1081	1543	Linalool	25.0
1115	1516	Camphor	3.4
1129	1668	*trans*-Verbenol	0.1
1142	1667	*δ-*Terpineol	3.0
1142	1693	Borneol	6.4
1158	1595	Terpinen-4-ol	1.4
1166	1692	α-Terpineol	4.5
1178	1780	Myrtenol	0.1
1210	1764	Citronellol	0.2
1233	1842	Geraniol	0.4
1266	1574	Bornyl acetate	0.3
1359	1755	Geranyl acetate	0.1
1382	1585	β-Elemene	0.3
1411	1590	*trans*-β-Caryophyllene	0.5
1498	1751	*γ-*Cadinene	0.1
1526	2070	Elemol	0.1
1557	1968	Caryophyllene oxide	0.4
1569	2072	Viridiflorol	0.2
1579	2025	Ledol	0.1
1615	2153	T-Cadinol	t
1628	2218	α-Cadinol	t
1628	2208	α-Eudesmol	t
		Monoterpene hydrocarbons	9.5
		Oxygen containing monoterpenes	85.5
		Sesquiterpene hydrocarbons	0.9
		Oxygen containing sesquiterpenes	0.9
		Total	96.8

RI^a^, retention indices on the SPB-1 column relative to C_8_ to C_24_ *n*-alkanes. RI^p^, retention indices on the SupelcoWax-10 column.

^a^Compounds listed in order of their elution on the SPB-1 column; t, traces (≤ 0.05%).

**Figure 1:**
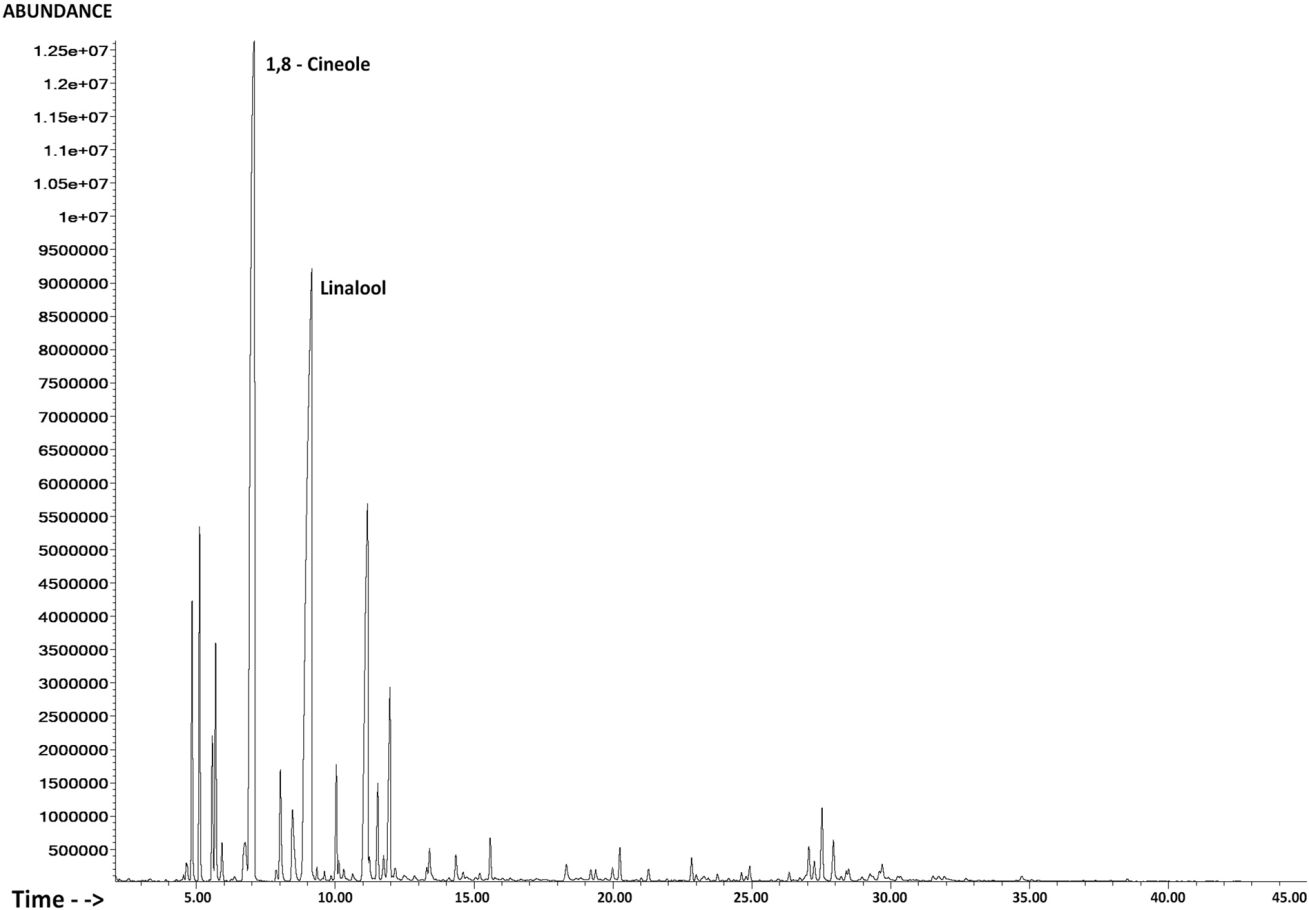
Total ion current chromatogram of the essential oil of *Thymus albicans.*

Oxygen containing monoterpenes represented the major fraction of the oil (85.5%). The major compounds identified were 1,8-cineole (40.5%) and linalool (25.0%), followed by borneol (6.4%) and α-terpineol (4.5%). The Total Ion Current (TIC) chromatogram, processed using HP Enhanced ChemStation software, is shown in Fig. 1. Our results are in accordance with the works of Morales Valverde^10^ and Salgueiro, et al.^35^ that pointed out 1,8-cineole as the major compound of *T. albicans* essential oil from Portugal followed by linalool. Miguel, et al.^36^ studied the oils obtained from leaves and from flowers, distinguishing the chemotypes 1,8-cineole and 1,8-cineole/linalool.

*Thymus albicans* essential oil, in the present study, was obtained from the flowering aerial parts with a yield of 2.4% (v/w). Our results are in agreement with the study of Aazza, et al.^21^ reporting a yield of 2.2%. This yield is quite relevant since it is higher than that obtained with industrially relevant species, such as *T. vulgaris*, *T. zygis* and *T. mastichina*, thus highlighting the industrial potential of *T. albicans*.

### Fungal growth (MIC and MLC)

As far as it is known, this is the first report on the antifungal effect of *T. albicans* essential oil. The oil showed some degree of inhibition against all *Candida*, *Cryptococcus neoformans*, dermatophytes and *Aspergillus* strains tested (Table 2), thus showing a broad-spectrum antifungal effect. Moreover, for the majority of yeast and all dermatophyte strains, MIC values were similar to MLC ones, pointing out a fungicidal effect of the oil. The highest antifungal activity was observed against *Trichophyton rubrum* CECT 2794 and *Epidermophyton floccosum* FF9 (MIC and MLC of 0.64 μL/mL).

**Table 2:** Antifungal activity (MIC and MLC) of *Thymus albicans* essential oil and its major compounds (1,8-cineole, linalool, borneol and α-terpineol) against collection type and clinical strains of *Candida*, *Cryptococcus neoformans*, dermatophytes and *Aspergillus.*

Strains	*Thymus albicans*		1,8-Cineole		Linalool		Borneol		α-Terpineol	
	MIC^a^	MLC^a^	MIC^a^	MLC^a^	MIC^a^	MLC^a^	MIC^a^	MLC^a^	MIC^a^	MLC^a^
*Candida albicans* ATCC 10231	1.25	2.5	10	10	5	5	2.5	> 20	1.25	2.5
*Candida tropicalis* ATCC 13803	2.5	2.5	20	20	5	5	2.5	> 20	1.25	2.5
*Candida krusei* H9	2.5	2.5	10	10	10	10	5	> 20	1.25	2.5
*Candida guilliermondii* MAT23	1.25	1.25	10	10	5	10	2.5	2.5	1.25	1.25–2.5
*Candida parapsilosis* ATCC 90,018	2.5	2.5	10	10	10	10	5	> 20	0.64–1.25	2.5
*Cryptococcus neoformans* CECT 1078	1.25	1.25	5–10	10	5	5	1.25	1.25	0.64–1.25	1.25
*Epidermophyton floccosum* FF9	0.64	0.64	5	5	1.25–2.5	2.5	2.5	2.5	1.25	1.25
*Microsporum canis* FF1	1.25	1.25	5	5	2.5	2.5	2.5	2.5	1.25	1.25
*Microsporum gypseum* CECT 2908	1.25	1.25	5–10	10	1.25–2.5	2.5	2.5	2.5	1.25	1.25
*Trichophyton mentagrophytes* FF7	1.25	1.25	5	5	1.25	2.5	2.5	5	1.25	2.5
*Trichophyton mentagrophytes* var. *interdigitale* CECT 2958	1.25	1.25	10	10	2.5	2.5–5	2.5	5	0.64	1.25
*Trichophyton rubrum* CECT 2794	0.64	0.64	2.5–5	5	1.25	1.25–2.5	2.5	2.5	1.25	1.25–2.5
*Trichophyton verrucosum* CECT 2992	1.25	1.25	10	10–20	1.25–2.5	1.25–2.5	2.5	2.5	0.64	1.25
*Aspergillus niger* ATCC 16404	2.5	10–20	10	> 20	5	≥ 20	5	> 20	0.64	10–20
*Aspergillus fumigatus* ATCC 46645	2.5	5	10	10–20	2.5	20	2.5	> 20	0.32–0.64	2.5
*Aspergillus flavus* F44	5	5–10	20	20	10	≥ 20	5	> 20	0.64–1.25	2.5

^a^MIC and MLC determined by macrodilution method and expressed as μL/mL.

Other essential oils with high amounts of 1,8-cineole, such as the ones isolated from *Salvia fruticosa, Thymus capitellatus and Thymus mastichina,* were previously shown to exert antifungal activity against several fungal species^16,37,38^.

Regarding the antifungal activity of *T. albicans* major compounds, 1,8-cineole, linalool, borneol and α-terpineol, only the later showed a slightly stronger effect than the essential oil. Among the strains tested, *C. parapsilosis* ATCC 90018, *Cryptococcus neoformans* CECT 1078 and *Aspergillus* strains were the most susceptible to α-terpineol with MIC values ranging from 0.32 to 1.25 μL/mL. Also, Hammer, et al.^39^ demonstrated that α-terpineol was more active than 1,8-cineole against some of the fungi species tested, namely *C. albicans*, *C. parapsilosis*, *E. floccosum*, *M. canis*, *T. mentagrophytes* var. *interdigitale*, *T. rubrum*, *A. niger*, *A. fumigatus* and *A. flavus.* Although 1,8-cineole had a weak activity, several studies reported synergistic effects of this compound with other monoterpenes^40^. Moreover, this compound has also shown synergistic effects with chlorhexidine, a commonly used antiseptic^41,42^.

### *Candida albicans* virulence factors

Among *Candida* species, *C. albicans* is the main etiological agent of candidiasis and one of the main causes of hospital-acquired infections^43^. The pathogenicity of *C. albicans* is associated with two major virulence factors: germ tube and biofilm formation. The germ tube (filamentation) confers increased resistance to phagocytosis and allows the penetration into deeper layers of the mucosa leading to invasive infections^44^. Biofilms, formed on both implanted medical devices and host tissues, potentiate *C. albicans* dissemination and increase its resistance to conventional drugs^45^. They act as reservoirs of multi-resistant yeast cells, which upon filamentation trigger resistant systemic infections. Despite the major role of these virulence factors in the pathogenesis of invasive *Candida* infections, to date, no-specific germ tube and biofilm antifungal drugs are available.

### *Candida albicans* germ tube formation

**Figure 2:**
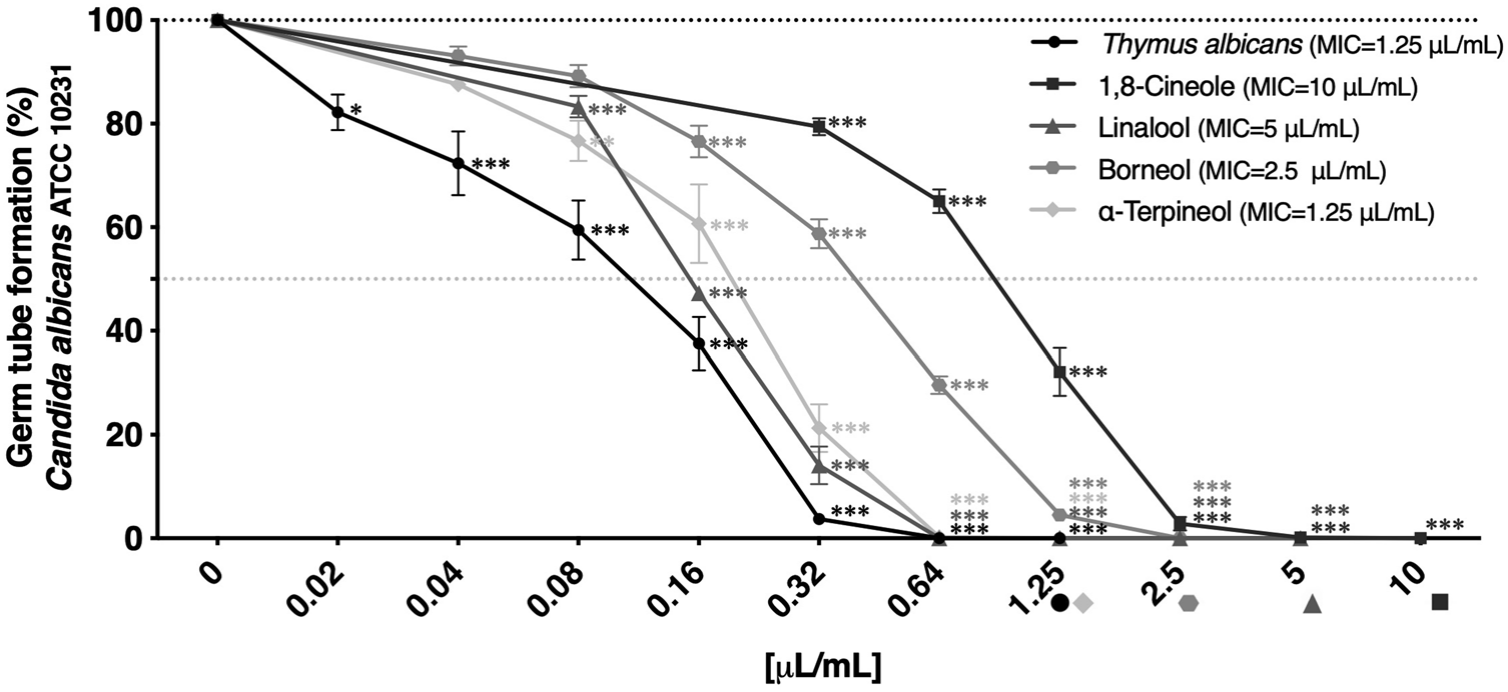
Germ tube formation in *Candida albicans* ATCC 10231 treated with sub-inhibitory concentrations—ranging from MIC to MIC/64—of *Thymus albicans* essential oil and its main compounds (1,8-cineole, linalool, borneol and α-terpineol)*.* The values are presented as percentage of control (oil-free samples with 1% DMSO (v/v)) ± SEM of at least three independent experiments performed in duplicate. Statistical differences were calculated by one-way ANOVA followed by Dunnett’s post hoc test (*p < 0.05, **p < 0.01, ***p < 0.001).

In our study, *T. albicans* essential oil was able to strongly inhibit the germ tube formation of *C. albicans* at concentrations much lower than the respective MIC. At 0.08 μL/mL the oil inhibited germ tube formation by 40.5% and at 0.32 μL/mL a 96.3% inhibition was attained (Fig. 2). These results are quite interesting since fluconazole, a conventional antifungal drug widely used in the clinic, despite inhibiting fungal growth at very low concentrations, is ineffective in decreasing germ tube formation. Indeed, even at concentrations up to 200 µg/mL the percentage of inhibition did not exceed 10% (Figure S1). Conversely, *T. albicans* essential oil was able to strongly reduce *C. albicans* germ tube formation at concentrations as low as 0.08 µL/mL.

Regarding the major compounds tested, only linalool could completely inhibit germ tube formation at sub-MIC concentrations (0.64 μL/mL). Hsu et al.^46^ and Zore et al.^47^ also reported the ability of linalool to decrease *C. albicans* germ tube formation at sub-inhibitory concentrations. The results obtained for 1,8-cineole are consistent with those reported by Pina‐Vaz et al.^16^. Moreover, in the same study, phenolic thyme essential oils from *T. vulgaris* and *T. zygis* were less effective than *T. albicans* in inhibiting germ tube formation, although both oils showed better antifungal activity (lower MIC values) against *C. albicans.*

### Disruption of preformed biofilms in *Candida albicans*

**Figure 3:**
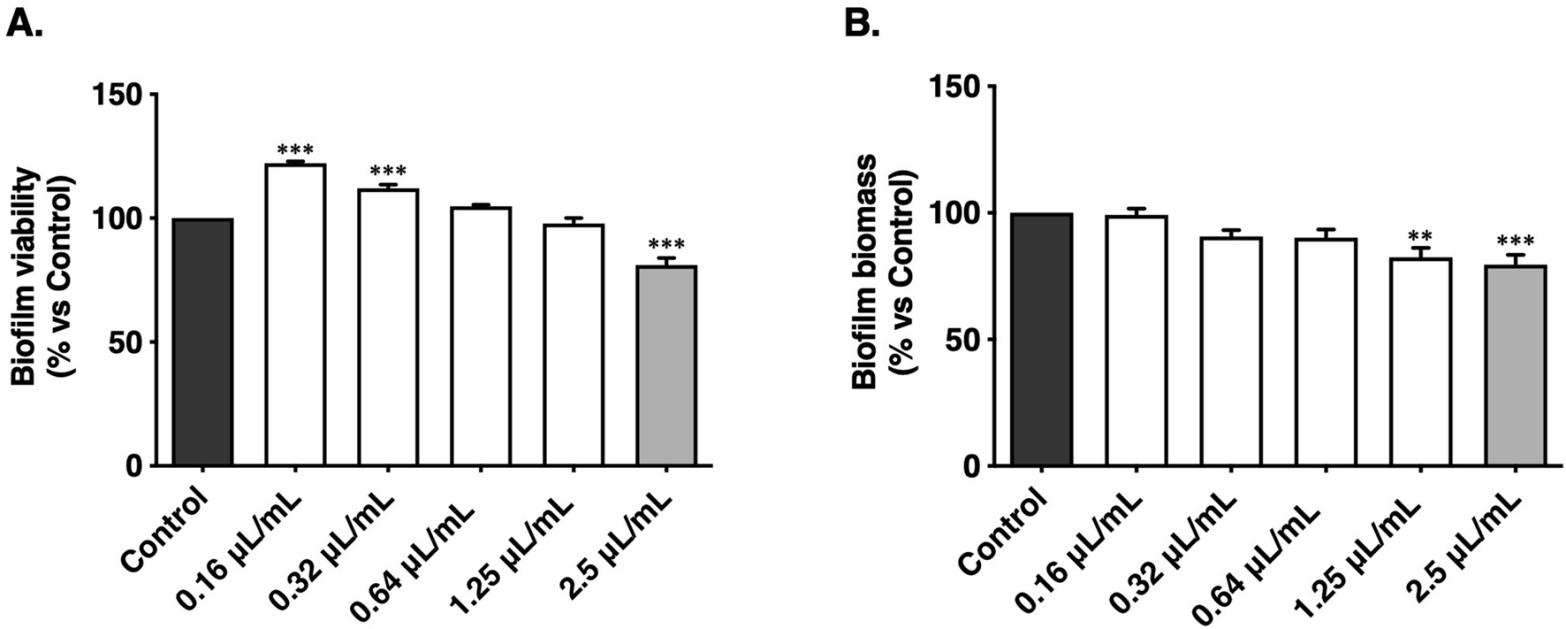
Influence of *Thymus albicans* essential oil on *Candida albicans* ATCC 10231 biofilm viability (**A**) and effect on biofilm biomass after 24 h (**B**). Each value represents the mean ± SEM of at least three independent experiments performed in duplicate. Statistical differences were calculated by one-way ANOVA followed by Dunnett’s post hoc test (**p < 0.01, ***p < 0.001). The grey bar represents the minimum lethal concentration (MLC) of *Thymus albicans* essential oil against *Candida albicans* ATCC 10231.

Besides germ tube formation, the pathogenicity of *C. albicans* is also associated with biofilm formation. In the present study, *T. albicans* essential oil was assessed for its capacity to decrease biofilm mass and viability (Fig. 3). At MIC values, the oil decreased biofilm mass, while fluconazole had no effect.

The main compound of *T. albicans* essential oil, 1,8-cineole, was shown to inhibit *C. albicans* biofilms at higher concentrations than the oil^41^. Also, other major compounds, namely linalool, borneol and α-terpineol were previously reported to disrupt *C. albicans* biofilm formation^46,48,49^.

Germ tube formation and biofilm integrity in *C. albicans* are important virulence factors that allow the transition from a superficial mycosis to a systemic infection. Overall, our results show that *T. albicans* essential oil presents antivirulent potential against *C. albicans* since it was able to inhibit both of these features. The possibility of targeting virulence factors at non-growth inhibitory concentrations is a promising therapeutic strategy, particularly because it may reduce the toxicity of antifungal therapy simultaneously reducing not only the selective pressure for the emergence of resistance among pathogenic fungi but also the impact on the host’s natural microbiota^44^.

### Anti-inflammatory activity

LPS-stimulated mouse macrophages (RAW 264.7) were used as an in vitro model of acute inflammation. Briefly, LPS, a *Toll-like* receptor 4 agonist and a component of the outer membrane of Gram-negative bacteria, acts upstream of the NF-κB signaling pathway inducing the phosphorylation and degradation of its inhibitory protein κB (IκB). Once released from inhibition, NF-κB translocates into the nucleus activating the transcription of genes coding for the inducible form of nitric oxide synthase (iNOS) and cyclooxygenase-2 (COX-2), two pro-inflammatory enzymes, among others. iNOS catalyzes the synthesis of NO through oxidation of L-arginine and COX-2 the conversion of arachidonic acid to prostaglandin E2 (PGE2)^50^.

In our study, the levels of NO stable metabolites (nitrites) before and after *T. albicans* oil treatment were measured by the colorimetric Griess reaction and used as a parameter to determine the anti-inflammatory potential of the essential oil.

**Figure 4:**
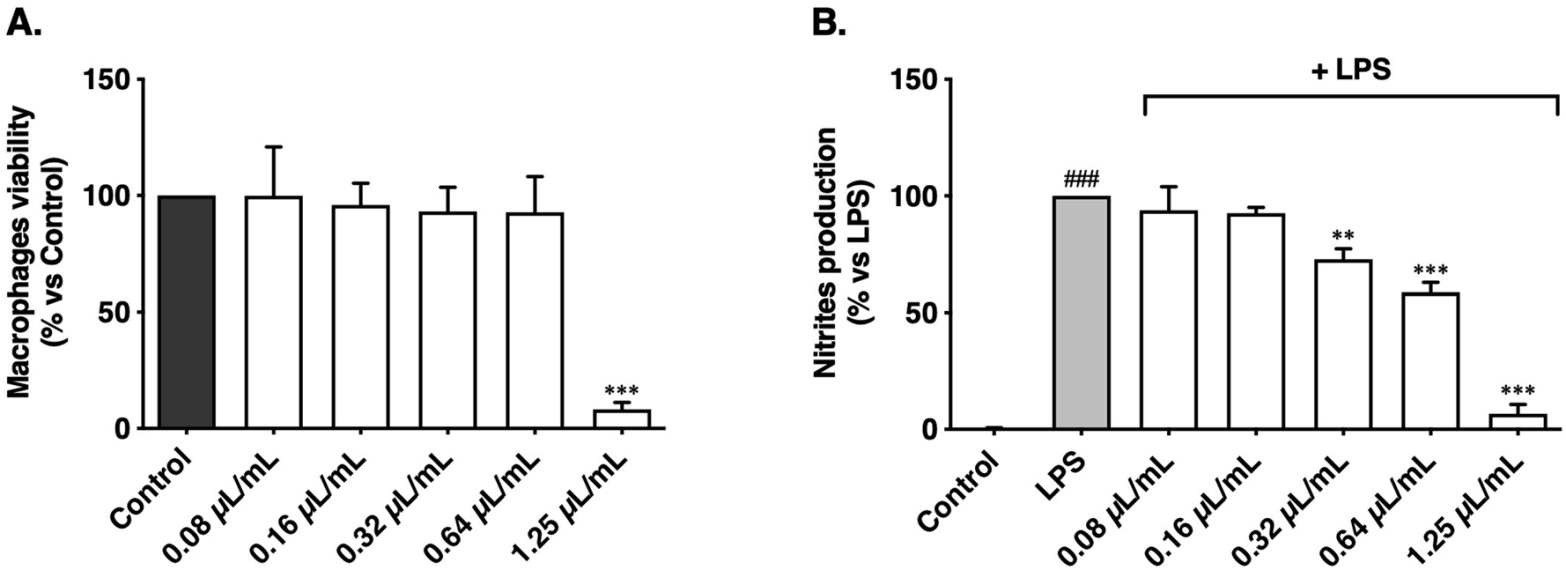
Effect of *Thymus albicans* essential oil on macrophages viability (**A**) and nitrites production (**B**). Cell viability is expressed as a percentage of MTT reduction in comparison to control cells (100% viability). Nitrite production is expressed as a percentage of nitrite production in comparison to cells stimulated with LPS alone (100% nitrite production). Each value represents the mean ± SEM from three experiments, performed in duplicate. Statistical differences between groups were calculated by one-way ANOVA followed by Dunnett’s post hoc test (###*p* < 0.001, compared to control (**B**); **p < 0.01, ***p < 0.001 compared to control (**A**) and to LPS (**B**)).

Our results demonstrate that *T. albicans* oil was able to inhibit nitrites production by LPS-stimulated macrophages in a dose-dependent manner. Importantly, a significant reduction by 27.16 and 41.32%, was observed at the non-cytotoxic concentrations of 0.32 and 0.64 μL/mL, respectively, in comparison with the LPS stimuli (Fig. 4). In an analogous experiment, Zuzarte et al.^51^ evaluated the effect of 1,8-cineole on the same parameter, demonstrating that it also inhibited the production of nitrites, however at higher concentrations (2.25 μL/mL). This indicates that other compounds may contribute to the anti-inflammatory activity of *T. albicans* oil.

### Mechanism of action: scavenging capacity or gene expression regulation?

**Figure 5:**
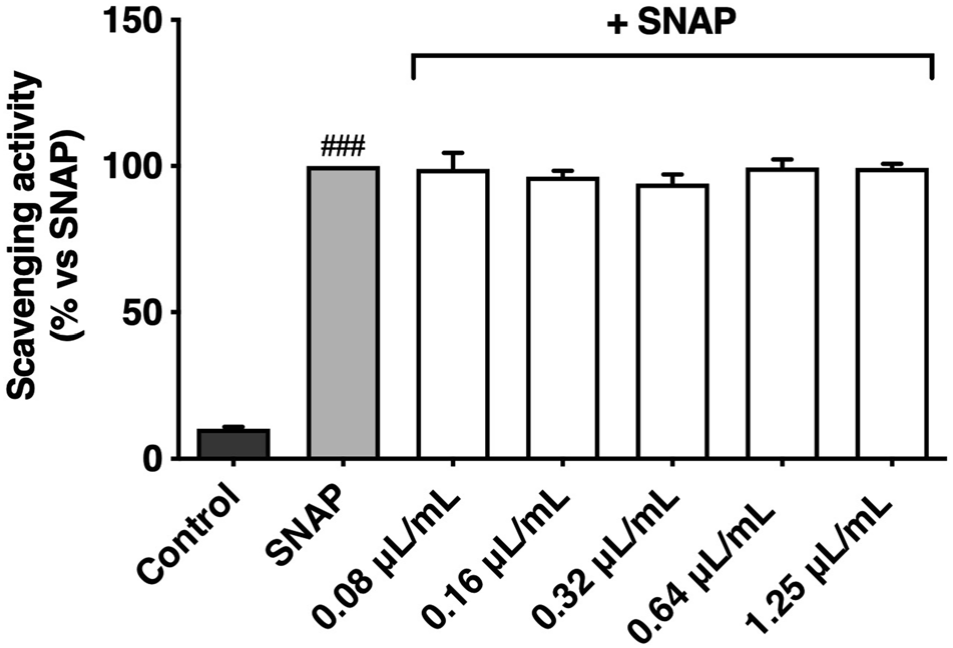
NO scavenging potential of *Thymus albicans* essential oil*.* Different concentrations of essential oil (0.08–1.25 μL/mL) were incubated with SNAP (300 μM) in culture medium for 3 h. Results are expressed as a percentage of NO release triggered by SNAP. Each value represents the mean ± SEM of three independent assays, performed in duplicate. Statistical differences were calculated by one-way ANOVA followed by Dunnett’s post hoc test (###*p* < 0.001, compared to control).

To clarify whether the lower levels of nitrites observed after *T. albicans* essential oil treatment were due to its direct scavenging potential, a reaction between a NO donor (SNAP) and different concentrations of the oil was performed. Since *T. albicans* essential oil failed to scavenge NO (Fig. 5), we could exclude this hypothesis to justify the reduction of nitrite levels in LPS-stimulated macrophages. In the study of Aazza, et al.^21^, *T. albicans* essential oil was shown to scavenge NO in vitro, however at much higher concentrations than those tested in our study.

**Figure 6:**
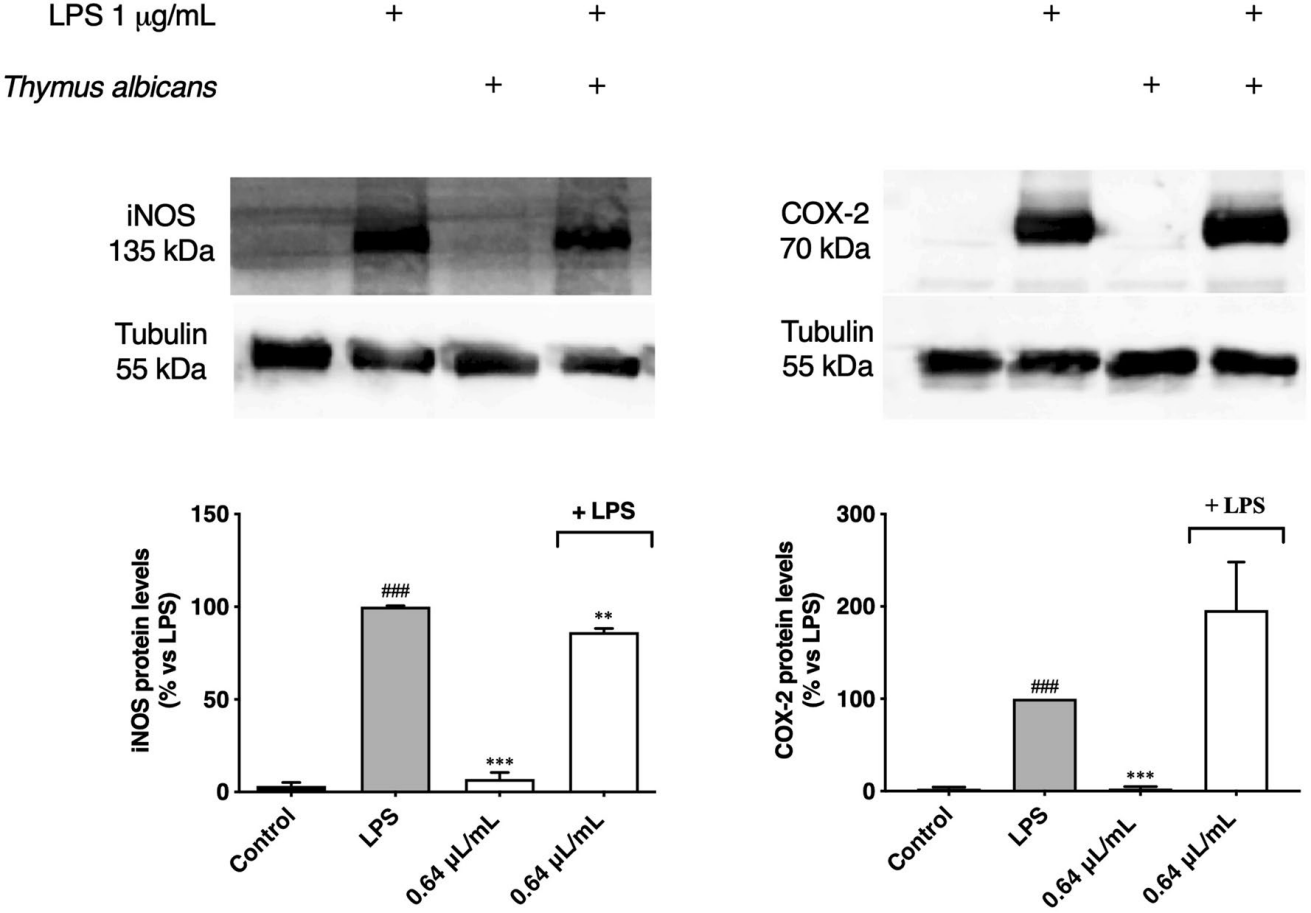
Effect of *Thymus albicans* essential oil on the iNOS and COX-2 protein levels in LPS stimulated RAW 264.7 macrophages. Cells (1.2 × 10^6^ cells/well) were kept 24 h in medium (control) or pre-treated for 1 h with 0.64 μL/mL of *T. albicans* essential oil and then stimulated with LPS (1 μg/mL). Total cell lysates were analyzed by western blot. Results are expressed as protein levels compared to LPS. Statistical differences were calculated by two-tailed unpaired student’s *t*-test (###*p* < 0.001, compared to control; **p < 0.01, ***p < 0.001, compared to LPS).

Another mechanism by which *T. albicans* essential oil could reduce the nitrite levels is the modulation of genes coding for key enzymes along the inflammatory pathway, namely iNOS and COX-2. Our results show that the expression of both enzymes by LPS-stimulated macrophages was significantly higher (*p* < 0.001) in comparison to the LPS-free control (Fig. 6), demonstrating the ability of LPS to induce the expression of these enzymes in our inflammatory-like model. Cells treated only with essential oils revealed neglectable levels of iNOS and COX-2 expression, ruling out possible pro-inflammatory effects of the oil.

At 0.64 μL/mL, *T. albicans* essential oil inhibited by 13.64% iNOS expression in comparison to the LPS stimulus (*p* < 0.01). This slight decrease of iNOS protein levels in LPS-stimulated macrophages pre-treated with *T. albicans* essential oil does not reflect the significant NO reduction evaluated through Griess reaction. It seems that *T. albicans* essential oil may exert its anti-inflammatory activity also by modulating iNOS activity or by inhibiting other signaling pathways. Further studies should be performed to deeply explore this hypothesis. Regarding COX-2 levels, no significant result was observed, indicating a selective effect of the oil towards iNOS expression (Fig. 6). Uncropped blots are provided in Supplementary Information (Fig. S2).

The anti-inflammatory activity of *T. albicans* main compounds is broadly reported in both pre-clinical and clinical studies. Accordingly, in a clinical study conducted by Juergens et al.^52^, 1,8-cineole revealed anti-inflammatory activity in patients with severe asthma. Using human cell lines, Greiner et al.^53^ demonstrated the ability of 1,8-cineole to attenuate the activity of NF-κB, thus decreasing its nuclear translocation and consequently reducing the expression of its target genes coding for pro-inflammatory mediators. Nogueira et al.^54^ showed that it modulates the production of pro-inflammatory cytokines by LPS-stimulated macrophages by interfering with NF-κB, p38 or ERK/MAPK pathways. Peana et al.^55^ demonstrated the capacity of linalool to reduce NO production in LPS-stimulated macrophages, although it failed to inhibit iNOS expression. The authors suggested that linalool could possibly act as a scavenger of NO or as an inhibitor of iNOS activity.

Huo et al.^56^ also showed the anti-inflammatory activity of linalool using in vitro and in vivo models. Linalool inhibited NF-kB and MAPK activation in LPS-stimulated macrophages and protected mice against LPS-induced acute lung injury by reducing the production of TNF-α and IL-6. Borneol was shown to decrease the expression of cytokines (IL-6 and IL-8) in human gingival fibroblasts^57^. These studies confirm the contribution of 1,8-cineole, linalool, borneol and α-terpineol to the anti-inflammatory activity of *T. albicans* essential oil.

Other thyme essential oils rich in 1,8-cineole were previously reported to have anti-inflammatory potential, namely *T. mastichina*, which was shown to inhibit lipoxygenase activity^21,29^, *T. hyemalis,* which reduced the secretion of pro-inflammatory cytokines (TNF-α, IL1β, and IL-6) in an inflammatory model of human macrophages stimulated with Cu^2+^-oxidized LDLs^58^, and *T. camphoratus* (chemotype 1,8-cineole/borneol) that inhibited both iNOS and COX-2 in the same model of LPS-stimulated macrophages used in this study^59^.

## Conclusion

*Thymus albicans* essential oil showed a broad spectrum antifungal activity. The oil was able to inhibit germ tube formation and biofilm integrity in *Candida albicans*, which are two key virulent factors of this highly prevalent etiologic agent of topical and invasive candidiasis in humans. In addition, at concentrations devoid of toxicity, *Thymus albicans* essential oil was able to reduce the production of the pro-inflammatory mediator NO. This anti-inflammatory effect was shown to be partly related to the reduction of iNOS levels.

The combination of antifungal and anti-inflammatory effects together with the high essential oil yield (2.4%) and absence of cytotoxicity at bioactive concentrations demonstrate the therapeutic potential of *Thymus albicans,* an endangered Iberian species. Moreover, the results obtained in the present study corroborate the traditional use of *Thymus* species as antimicrobial and anti-inflammatory agents.

Also, *T. albicans* revealed higher essential oil yield than other widely marketed thyme species like *Thymus vulgaris* L., *Thymus zygis* L. and *T. mastichina*. It is important to underline that although *T. albicans* and *T. mastichina* are both species of the *Mastichina* section with high content of 1,8-cineole, only *T. mastichina* oil has industrial relevance, proven by the established ISO 4728:2003 standard^15^. Indeed, the bioactivities herein reported confirm the potential of *T. albicans* essential oil paving the way for further studies on the pharmaceutical applications of this species, which might contribute to improve its conservation status.

## Material and methods

### Plant material

Flowering parts of *Thymus albicans* Hoffmanns. and Link were collected at the same time in the area of Foz de Almargem coastal lagoon, Algarve, Portugal. All plants presented a similar stage of development. Voucher specimens were included in the Herbarium of the University of Coimbra (COI), with the accession number Moura 4796. Dr. Jorge Paiva, a taxonomist at the University of Coimbra, confirmed species authenticity and plant names were checked with https://www.theplantlist.org.

### Essential oil isolation and analysis

The essential oils from the aerial parts of *T. albicans* plants were obtained by hydrodistillation for 3 h, using a Clevenger-type apparatus^60^. Chemical analyses were carried out by both gas chromatography (GC-FID) and gas chromatography-mass spectroscopy (GC–MS) using fused silica capillary columns with two stationary phases (SPB-1 and SupelcoWax 10) as previously reported^61^.

The compounds were identified based on their GC retention indices on both columns and through their mass spectra. Retention indices, calculated by linear interpolation relative to the retention times of C_8_–C_24_ *n*-alkanes (Eq. 1)^62^, were compared with those of authentic products included in the Faculty of Pharmacy of the University of Coimbra laboratory database and/or literature data^63,64^. Acquired mass spectra were compared with reference spectra from the laboratory database, Wiley/NIST library and literature data^63,65^. Relative amounts of individual components were calculated based on GC raw data areas without a response factor correction for flame ionization detection.

1 $$I_{a}^{\varphi } = 100z + 100\left[ {\frac{{T_{\left( a \right)} - T_{\left( z \right)} }}{{T_{{\left( {z + 1} \right)}} - T_{\left( z \right)} }}} \right]$$

*Iφ*, experimental retention indices of the analyte “a” in the stationary phase “*φ*”; *z*, number of carbons of the *n*-alkane with the closest previous elution to “*a*”; *T*_(*z*)_, retention time of the *n*-alkane with the closest previous elution to “*a*”; *T*_(*z*+1)_, retention time of the* n*-alkane with the closest after elution to “*a*”; *T*_(*a*)_, Retention time of the analyte “*a*”*.*

### Pure and reference compounds

The following synthetic compounds were purchased: 1,8-cineole (extra pure, Merck, Darmstadt, Germany), linalool (pure, Fluka, Steinheim, Germany), borneol (pure, Fluka, Steinheim, Germany) and α-terpineol (pure, Extrasynthese, Genay, France).

### Fungal growth (MIC and MLC)

The antifungal activity of *T. albicans* essential oil and its major compounds (1,8-cineole, linalool, borneol and α-terpineol) was evaluated against two *Candida* strains isolated from recurrent cases of vulvovaginal and oral candidiasis (*C. krusei* H9, *C. guilliermondii* MAT23) and three type strains (*C. albicans* ATCC 10231, *C. tropicalis* ATCC 13803, *C. parapsilosis* ATCC 90018); a *Cryptococcus neoformans* type strain (*C. neoformans* CECT 1078); three clinical dermatophyte strains isolated from nails and skin (*Epidermophyton floccosum* FF9, *Microsporum canis* FF1, *Trichophyton mentagrophytes* FF7) and four type strains (*M. gypseum* CECT 2908, *T. mentagrophytes* var. *interdigitale* CECT 2958, *T. rubrum* CECT 2794, *T. verrucosum* CECT 2992); one *Aspergillus* clinical strain isolated from bronchial secretions (*A. flavus* F44) and two type strains (*A. niger* ATCC 16404, *A. fumigatus* ATCC 46645). The fungal isolates were stored on Sabouraud broth with 20% of glycerol at − 80 °C and previously cultured on Sabouraud Dextrose Agar (SDA) or Potato Dextrose Agar (PDA) to ensure optimal growth characteristics and purity for the antifungal susceptibility testing.

Minimal Inhibitory Concentrations (MICs) and Minimal Lethal Concentrations (MLCs) were determined using the macrodilution method, according to Clinical and Laboratory Standards Institute guidelines for yeasts and filamentous fungi as described by Zuzarte et al.^51^.

### *Candida albicans* virulence factors

#### Germ tube inhibition

Cell suspensions (1.0 ± 0.2 × 10^6^ CFU/mL) from overnight SDA cultures were prepared in NYP medium [N-acetylglucosamine (Sigma-Aldrich, St. Louis, MO, USA; 10^–3^ mol/L), yeast nitrogen base (Difco, Detroit, MI, USA; 3.35 g/L), proline (Fluka; 1023 mol/L) and NaCl (4.5 g/L), pH 6.7 ± 0.1]. Serial two-fold essential oil dilutions were previously prepared in DMSO [maximum concentration of 1% (v/v)], and added to the inoculum suspensions to obtain a range of test concentrations from MIC value to MIC/64 (1.25–0.02 μL/mL). Oil-free controls with and without DMSO were included. After 3 h of incubation in a static chamber at 37 °C, homogenized cell suspensions of each treatment were mounted onto a Neubauer chamber; 100 cells per replica were counted and visually scored, excluding gemulating cells. The percentage of filamentation was defined as the number of cells in which the size of the germ tube was equal or bigger than the blastopore’s diameter.

#### Disruption of preformed biofilms

The effect of the *T. albicans* essential oil on the disruption of *C. albicans* ATCC 10231 preformed biofilm was investigated following the method described by Alves-Silva et al.^66^. Briefly, a cell suspension of *C. albicans* was prepared in Yeast Peptone Dextrose (YPD) broth (1% yeast extract, 2% peptone, and 2% dextrose) and incubated for 24 h at 37 °C. A final suspension of 1 × 10^6^ CFU/mL was added to 96-well microtiter plates and incubated for 24 h at 37 °C, to form the biofilms. After three washing steps with PBS, the essential oils (0.16–2.5 µL/mL) prepared in RPMI were added and incubated for 24 h at 37 °C. Negative and positive controls were included using sterile and inoculated RPMI media, respectively.

### Anti-inflammatory activity

#### Cell line and culture conditions

The mouse macrophage cell line, RAW 264.7 (ATCC-TIB-71) was cultured in Dulbecco’s modified eagle medium (DMEM) (Invitrogen, California, USA) supplemented with 10% (v/v) of non-inactivated fetal bovine serum (Invitrogen), 3.02 g/L sodium bicarbonate (Sigma), 100 μg/mL streptomycin (Sigma) and 100 U/mL penicillin (Sigma). The cells were maintained at 37 °C in humidified atmosphere with 5% CO_2_ and 95% air. Adhesion, growth and morphological patterns were monitored by optical microscopy. Cells were regularly sub-cultured every two–three days and kept in culture for a maximum of three months.

#### Cell viability

The effect of *T. albicans* essential oil on macrophages viability (0.3 × 10^6^ cells/well of 48-well plates) was evaluated by MTT assay, as described by Zuzarte et al.^59^.

#### Nitric oxide production

The effect of the essential oil on NO production was evaluated by quantifying the concentration of its stable metabolites (nitrites) in macrophages culture supernatants, using the Griess colorimetric reagent, following the protocol described by Zuzarte et al.^59^.

### Essential oil’s mechanism of action in LPS-stimulated macrophages

#### Nitric oxide scavenging potential

The NO-scavenging potential of *T. albicans* essential oil was evaluated using S-nitroso-N-acetyl-DL-penicillamine (SNAP) reagent as a NO donor. The essential oil dilutions in culture medium were incubated with 300 μM SNAP for 3 h in the dark at room temperature. An oil free control was included. Nitrite quantification was performed as described previously for nitric oxide production.

#### Expression of inducible nitric oxide synthase and cyclooxygenase-2

Western blot analyses were performed to evaluate the influence of the essential oil on the expression of inducible nitric oxide synthase (iNOS) and cyclooxygenase-2 (COX-2) on LPS-stimulated macrophages. Total cell lysates, protein concentration determination and western blot analysis were performed according to Zuzarte et al.^59^. Membranes were revealed using a Typhoon FLA 9000 fluorescence reader (GE Healthcare, Chicago, IL, USA) and protein bands were measured by densitometric analysis using TotalLab TL120 (TotalLab, Newcastle, UK).

### Statistical analyses

The results are expressed as mean ± SEM of three independent experiments performed in duplicate. Differences between groups were calculated using one-way ANOVA followed by Dunnett’s post hoc test or t-test, using GraphPad Prism v7.0a for Mac OS X.

## Supplementary information

Supplementary Information.

## Data Availability

The data that support the findings of this study are available upon request from the corresponding author, MZ.
