# Supplementary material for: Antifungal and anti-inflammatory potential of the endangered aromatic plant Thymus albicans
Source: Sci Rep. 2020 Nov 2;10:18859. doi: 10.1038/s41598-020-75244-w (PMC7608662; doi:10.1038/s41598-020-75244-w)
Supplement: Supplementary file 1 — Supplementary Information. [file 41598_2020_75244_MOESM1_ESM.pdf]

Supplementary Information

Antifungal and anti-inflammatory potential of the endangered aromatic plant *Thymus albicans*

Mariana Roxo[1], Mónica Zuzarte[2,3,4*], Maria José Gonçalves[5,6], Jorge M. Alves-Silva[2,3,4,5], Carlos Cavaleiro[5,6], Maria Teresa Cruz[3,5,7], Lígia Salgueiro[5,6]

[1] Institute of Pharmacy and Molecular Biotechnology (IPMB), Heidelberg University, Heidelberg, Germany

[2] Faculty of Medicine, Coimbra Institute for Clinical and Biomedical Research (iCBR), University of Coimbra, Coimbra, Portugal

[3] Centre for Innovative Biomedicine and Biotechnology (CIBB), University of Coimbra, Coimbra, Portugal

[4] Clinical Academic Centre of Coimbra (CACC), Coimbra, Portugal

[5] Faculty of Pharmacy of the University of Coimbra, University of Coimbra, Coimbra, Portugal

[6] Chemical Process Engineering and Forest Products Research Centre (CIEPQPF), Department of Chemical Engineering, Faculty of Sciences and Technology University of Coimbra, Coimbra, Portugal

[7] Center for Neuroscience and Cell Biology (CNC), Coimbra, Portugal

Corresponding author: Mónica Zuzarte, mzuzarte@uc.pt, Faculty of Medicine, University of Coimbra, Azinhaga de S. Comba, 3000-354 Coimbra, Portugal. Tel: +351239480220

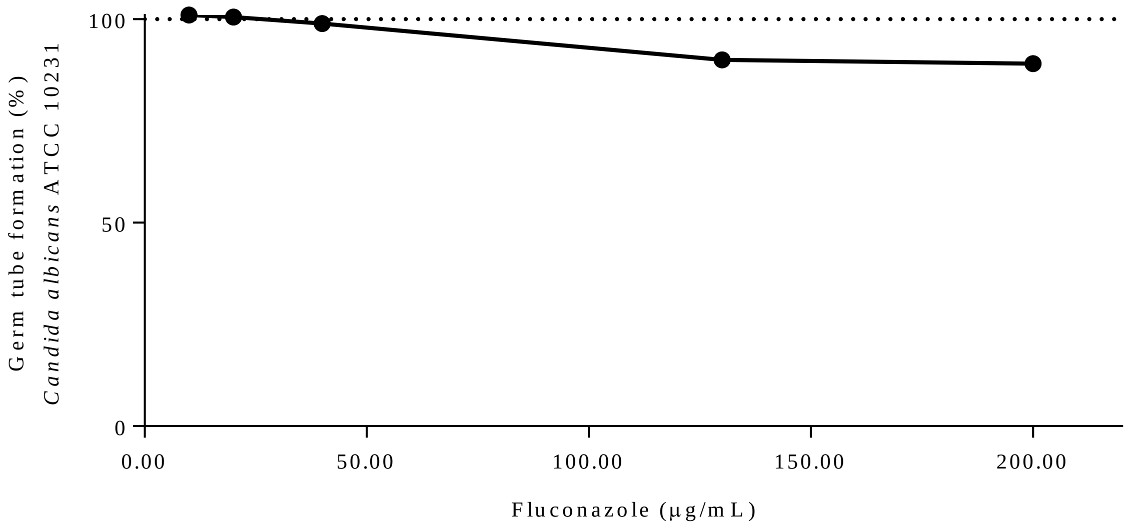

Figure S1. Germ tube formation in *Candida albicans* ATCC 10231 treated with fluconazole.

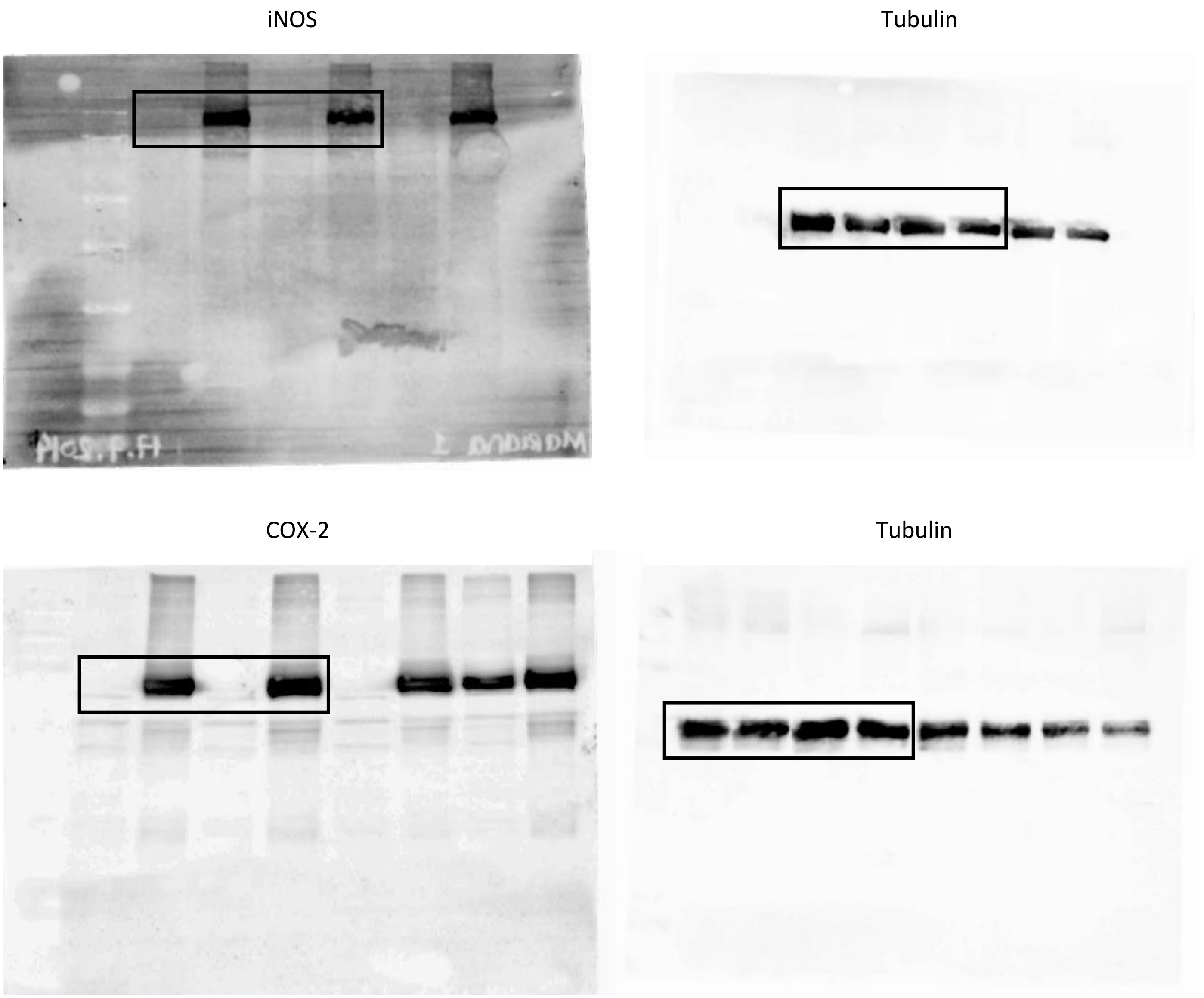

Figure S2. Original blots with cropped region marked.
